# *SUBGROUPS*: a computer tool at the Bilbao Crystallographic Server for the study of pseudo-symmetric or distorted structures

**DOI:** 10.1107/S1600576724008070

**Published:** 2024-10-01

**Authors:** Emre S. Tasci, Luis Elcoro, J. Manuel Perez-Mato, Gemma de la Flor, Mois I. Aroyo

**Affiliations:** a Physics Engineering Department, Hacettepe University, Ankara, Türkiye; b Departamento de Física, Facultad de Ciencia y Tecnología Universidad del Pais Vasco UPV/EHU Apartado 644 Bilbao Spain; c Facultad de Ciencia y Tecnología Universidad del Pais Vasco UPV/EHU Apartado 644 Bilbao Spain; d Institute of Applied Geosciences, Karlsruhe Institute of Technology, Karlsruhe, Germany; Tohoku University, Japan

**Keywords:** group–subgroup relations, pseudo-symmetry, distorted structures, group theory applications, computational crystallography

## Abstract

*SUBGROUPS* is a free online program at the Bilbao Crystallographic Server (https://www.cryst.ehu.es/). It permits the exploration of all possible symmetries resulting from the distortion of a higher-symmetry parent structure, provided that the relation between the lattices of the distorted and parent structures is known. The program calculates all the subgroups of the parent space group which comply with this relation. The required minimal input is the space-group information of the parent structure and the relation of the unit cell of the distorted or pseudo-symmetric structure with that of the parent structure. Alternatively, the wavevector(s) observed in the diffraction data characterizing the distortion can be introduced. Additional conditions can be added, including filters related to space-group representations. The program provides very detailed information on all the subgroups, including group–subgroup hierarchy graphs. If a Crystallographic Information Framework (CIF) file of the parent high-symmetry structure is uploaded, the program generates CIF files of the parent structure described under each of the chosen lower symmetries. These CIF files may then be used as starting points for the refinement of the distorted structure under these possible symmetries. They can also be used for density functional theory calculations or for any other type of analysis. The power and efficiency of the program are illustrated with a few examples.

## Abstract 2

Documentation is presented for the *SUBGROUPS* tool at the Bilbao Crystallographic Server for the exploration of all possible symmetries resulting from the distortion of a higher-symmetry parent structure (provided that the relation between the lattices of the distorted and parent structures is known).

## 1. Introduction

The determination of a distorted or pseudo-symmetric commensurate structure can be quite challenging. In such types of structure the diffraction data can be quantitatively explained to a good approximation by a structural model of higher symmetry, which we shall call the parent structure. The small symmetry-breaking deviations from this model generally only introduce very weak additional features that can manifest themselves in a wide range of properties which could be used to distinguish the actual symmetry of the crystal but, alas, these may be quite difficult to resolve and assess. Similar problems can happen in density functional theory (DFT) calculations when trying to determine the ground state of a material which deviates slightly from a parent structure of higher symmetry. In either of these two cases the parent structure may already be known as a virtual idealized arrangement for the family of materials to which the investigated material belongs, or it may be a real structure corresponding to a different phase of the same material where the symmetry-breaking distortion is not present. If a starting model for the parent structure is available (which is the case for these kinds of situation), as the deviations from this model are small, the structure determination can be reduced to a fit of the diffraction data through a refinement process, or in the case of DFT calculations to an energy minimization around the parent structure in configuration space. For these processes it is, however, convenient, or even in some cases necessary, to make a prior assumption on some specific space-group symmetry for the investigated structure, which by definition should be described by a subgroup of that associated with the parent structure. As a first step, it is then desirable to enumerate systematically all possible subgroups of the parent space group which may be consistent with the diffraction data and should be checked. We present here the online program *SUBGROUPS*, freely accessible at the Bilbao Crystallographic Server (https://www.cryst.ehu.es/) (Aroyo *et al.*, 2006*a* ▸,*b* ▸, 2011 ▸), which has been developed to perform this preliminary task. The program enumerates all possible symmetries as subgroups of the parent space group, with optional comprehensive information about them and their group–subgroup relationships. From a practical point of view, once the list of possible non-equivalent subgroup symmetries is provided, the program optionally generates appropriate Crystallographic Information Framework (CIF) files of any input parent structure with its symmetry reduced to each of these subgroups.

Some of the features of the present program are similar to those in *ISODISTORT* (Stokes *et al.*, 2016*b* ▸) and/or in *ISOSUBGROUP* (Campbell *et al.*, 2006 ▸) from the *ISOTROPY Software Suite* (Stokes *et al.*, 2016*a* ▸). However, the present program approaches the problem in a different way, as space-group representations are only considered optionally once the set of possible subgroups has been calculated. *SUBGROUPS* also has optional outputs, including graphs, which can be highly useful as complementary information. In the next two sections the main features of the program and its different outputs are explained. Some examples of application are then shown.

## 2. The program *SUBGROUPS*

The program *SUBGROUPS* is available in the *Group–Subgroup Relations of Space Groups* section of the Bilbao Crystallographic Server (BCS).

The mathematical procedures and algorithms used by the program are explained in detail in Appendix *A*[App appa], whereas in the text we mainly focus on the usage of the program and types of possible applications.

### 2.1. Basic input and output

The input of the program *SUBGROUPS* may seem somewhat complex at first glance, but it can be divided into two parts: (i) a minimal input required to run the program, and (ii) a set of optional filters (see Section 3), based on different criteria, which can refine and narrow down the results obtained in (i). As minimal input, *SUBGROUPS* only requires the parent space group and the relation of the lattice of the investigated structure with that of the parent structure to be specified. The determination of this lattice relation from the diffraction data of the structure is usually rather straight­forward. The symmetry of the distorted structure, as it is necessarily described by a subgroup of the parent space group, must have a lattice which, apart from a possible strain, must be either the same lattice or a sublattice. Its unit cell is then defined with respect to the parent unit cell as a *supercell*, which generates a subset of the parent lattice translations. In the simplest case where the parent lattice is mantained in the distorted structure, this supercell coincides with the parent unit cell.

The parent space group is introduced using its serial number according to *International Tables for Crystallography*, Vol. A (henceforth referred to as *IT*A) (Aroyo, 2016 ▸), and the program assumes that the space group and its unit cell are to be considered in the standard/default setting used in the BCS. This setting is the one found in *IT*A, but in the case of space groups which have more than one description in *IT*A, a fixed choice among them is made.^1^ The basis vectors which define the supercell of the distorted structure are introduced as linear combinations of the basis vectors of the conventional unit cell of the parent space group, with the possibility of adding some centring.

Alternatively, the lattice relation between the two structures can be introduced in reciprocal space, indicating the primary modulation wavevector or vectors present in the distortion. In the simplest case, if the lattice is maintained, this modulation vector should be null. In many cases it can be a single non-zero wavevector or a set of them (symmetry related or not by the operations of the parent space group). As the program is only intended for commensurate structures, the input wavevectors should be commensurate and described with respect to the conventional reciprocal unit cell of the parent space group. The subgroups sought must be compatible with these modulation wavevectors such that the product of each modulation vector with the translations forming the subgroup must be an integer. As we will see in Section 3.4, this alternative form of introducing the lattice of the distorted structure is especially convenient if one wants to restrict the possible symmetries, following Landau theory, to those that can result from a distortion transforming according to one or more specific irreducible representations of the parent space group.

The program calculates and lists all the possible subgroups of the parent space group which have the translation subgroup defined by the input supercell. The subgroups are classified according to *conjugacy classes* with respect to the parent space group, and the first output only lists one subgroup (chosen arbitrarily) as the representative of each conjugacy class (the process is explained in detail in Section 2.2). This first list can therefore be considered as an enumeration of the conjugacy classes describing all possible distinct compatible symmetries.

All subgroups within a conjugacy class are physically equivalent, *i.e.* they can be associated with physically equivalent domain-like distorted structures which are related by the lost operations. Therefore, for the purpose of enumerating distinct possible symmetries of the investigated structure, a single subgroup for each conjugacy class, as shown in this first list, is sufficient. However, if the program is used to identify the relation between a parent structure and a specific known distorted structure, then the space group used for the description of the distorted structure may not be the one chosen by the program as representative of the conjugacy class. In this case, the appropriate subgroup must be found within the list of conjugate subgroups, which can also be provided by the program (see Section 2.2).

**Figure 1:**
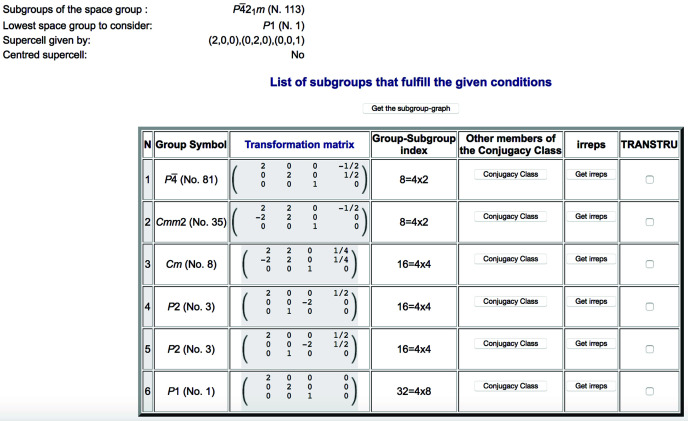
Possible symmetries, as obtained with *SUBGROUPS*, for a distorted structure having 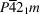 (No. 113) as its parent space group and with a primitive unit cell approximately given by 2**a**, 2**b**, **c** with respect to the parent unit cell. Only a representative subgroup for each conjugacy class of subgroups is listed. See the text for further explanations about each column in this table.

Fig. 1 ▸ shows as an example the list of conjugacy classes provided by the program for the parent space group 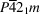 (No. 113) and a supercell defined by 2**a**, 2**b**, **c** with respect to the parent unit cell. Each listed subgroup is unambiguously defined by its space-group type in the second column and the choice of unit cell and origin (shown in the third column) which would transform the operations of this subgroup of the parent space group to the standard setting of the indicated space-group type. This transformation is described by a matrix–column pair (**P**, **p**) and consists of two parts: a *linear part***P** given by a (3 × 3) matrix, and an *origin shift***p** = (*p*_1_, *p*_2_, *p*_3_) given by a (3 × 1) column vector. This transformation (**P**, **p**) is defined with respect to the unit cell (**a**_p_, **b**_p_, **c**_p_) and origin **O**_p_ of the parent space group in the following form: 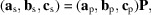

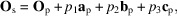
where (**a**_s_, **b**_s_, **c**_s_) and **O**_s_ are the unit-cell basis vectors and origin, respectively, for which the subgroup operations take the standard/default form available in the BCS and in *IT*A. This means that the transformed basis vectors are determined by the column coefficients of the matrix **P** (not by its row coefficients). This transformation to the standard setting is in general not unique and the program just makes a rather arbitrary choice among the possible ones. Hereinafter, specific transformations (**P**, **p**) are often written in the form (*P*_11_**a** + *P*_21_**b** + *P*_31_**c**, *P*_12_**a** + *P*_22_**b** + *P*_32_**c**, *P*_13_**a** + *P*_23_**b** + *P*_33_**c**; *p*_1_, *p*_2_, *p*_3_).

We stress that the space-group symbol in the second column in Fig. 1 ▸ is in general insufficient to define the subgroup. In most cases the transformation (**P**, **p**) is also necessary to eliminate any ambiguity. It is the application of the inverse of this transformation to the operations of the space-group type, expressed in its standard setting, which yields the set of operations of the parent space group (in its standard setting) constituting the defined subgroup. In fact, as shown in Fig. 1 ▸, there can be different subgroups belonging to different conjugacy classes which have the same space-group type, and it is only the transformation listed in the third column that distinguishes them.

**Figure 2:**
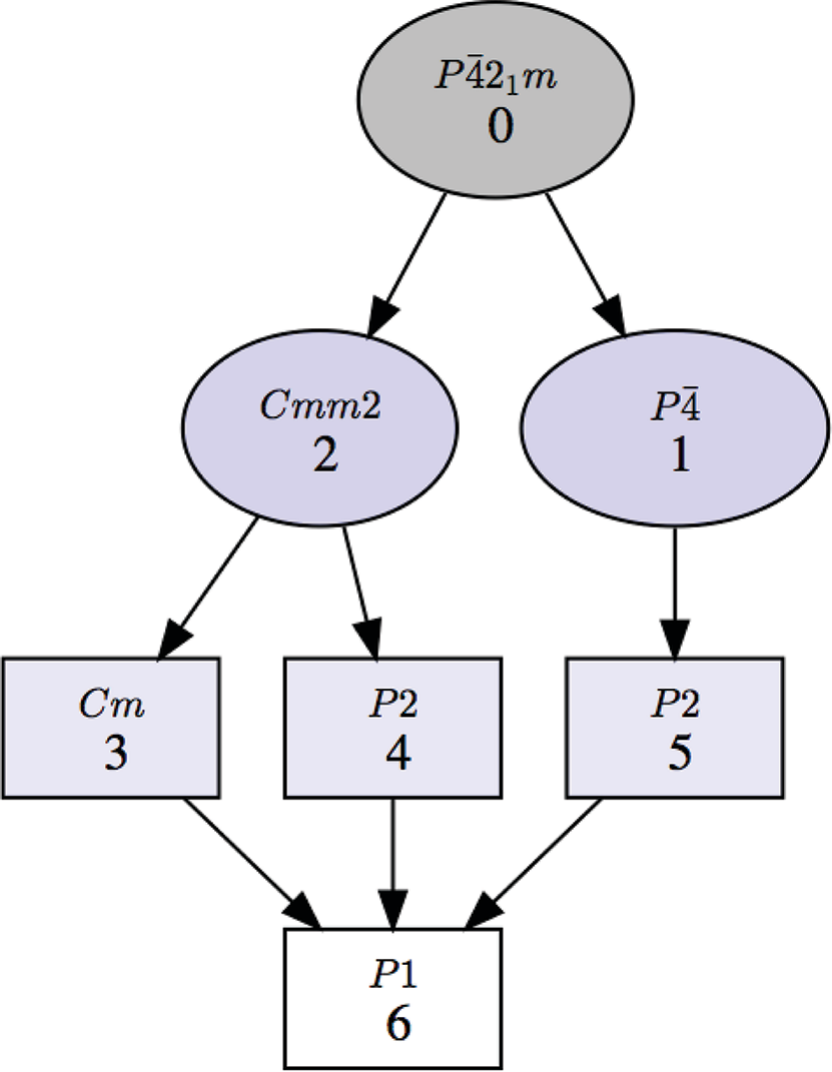
A group–subgroup graph for the symmetries shown in Fig. 1 ▸. The graph refers to conjugacy classes of subgroups rather than specific subgroups. The graph can be generated with or without the numerical labels given to the subgroups in the list shown in Fig. 1 (first column). The default option with these numerical labels is shown here.

The program can depict the group–subgroup hierarchy among the listed symmetries in the form of a graph, as shown in Fig. 2 ▸ for the list of subgroups in Fig. 1 ▸. We stress that this group–subgroup graph represents group–subgroup relations between unspecified subgroups belonging to the corresponding conjugacy classes. Therefore, in general they do not imply a group–subgroup relation between the specific subgroups that have been chosen as representatives in the accompanying list. It will be shown in Section 2.2 that detailed graphs of group–subgroup relations between specific subgroups can be obtained if the subgroups within each conjugacy class are listed. *SUBGROUPS* generates all these graphs using the open graph visualization system *Graphviz* (Gansner & North, 2000 ▸).

The fourth column in Fig. 1 ▸ indicates the subgroup index with respect to the parent group, *i.e.* the ratio between the number of operations in the parent space group and those in the subgroup (even though the number of operations in the groups can be infinite, their ratio is still well defined as the number of cosets in the decomposition of the parent space group with respect to the subgroup). This subgroup index gives the number of domain states that can be expected in a distorted phase with this symmetry. It is shown decomposed as the product of two factors, the first one relating the two lattices (*klassengleich* index) and the second one the two point groups (*translationengleich* index). The *klassengleich* index is determined by the lattice relation introduced by the user and therefore is the same for all listed subgroups. The *klassengleich* index determines the number of distinct domain states related by lost lattice translations (not distinguishable in diffraction experiments), while the *translationengleich* index gives the number of distinct orientation domain states associated with lost rotation, reflection, roto-inversion or inversion symmetry operations.

### 2.2. Detailed description of the subgroups forming each conjugacy class

**Figure 3:**
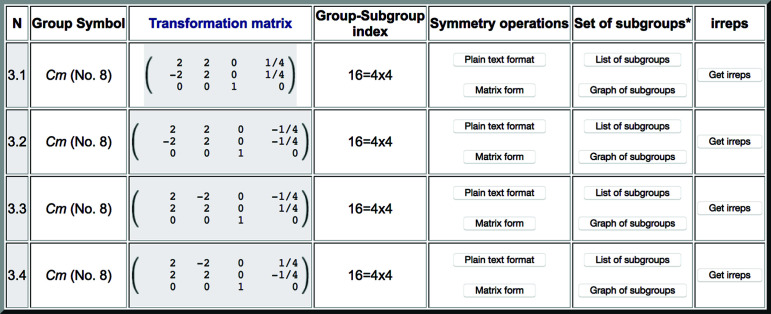
A list of subgroups of space group 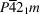 (No. 113) belonging to the conjugacy class of subgroups of type *Cm*, listed in the third row of Fig. 1. The first subgroup is the one used as a representative in the list of conjugacy classes in Fig. 1. See the text for an explanation of each column.

The first output of the program includes a link to detailed information on each conjugacy class (fifth column in Fig. 1 ▸). The subgroups belonging to the conjugacy class of which the chosen subgroup is a representative are enumerated. As an example, Fig. 3 ▸ shows the list obtained with this option for the conjugacy class with space-group type *Cm*, which is listed in Fig. 1 ▸. It is very important to stress that, in general, this list may not include the whole set of subgroups within the conjugacy class, as it is restricted to the conjugate subgroups that are compatible with the lattice relation introduced in the input. Conjugate subgroups belonging to the same conjugacy class, but with different orientations such that their supercell does not coincide with that of the input, or with symmetry-related rotated modulation wavevector(s) different from those introduced, are not listed by the program.

Inspecting the transformation to the standard setting of each subgroup in Fig. 3 ▸, one can see the orientation of its monoclinic axis with respect to the parent unit cell and the position of its standard origin, *i.e.* the position of the mirror plane within the parent structure. Thus the subgroups *Cm* are distinguished by either the location of the preserved mirror plane and/or its orientation. Two subgroups keep the mirror plane perpendicular to the direction [110], while the other two subgroups keep the mirror plane perpendicular to the direction 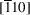, both directions being symmetry equivalent in the parent space group. A column with information on the index of the subgroup is also present in this table, but in this case it is redundant, as the index is necessarily the same for all conjugate subgroups.

**Figure 4:**
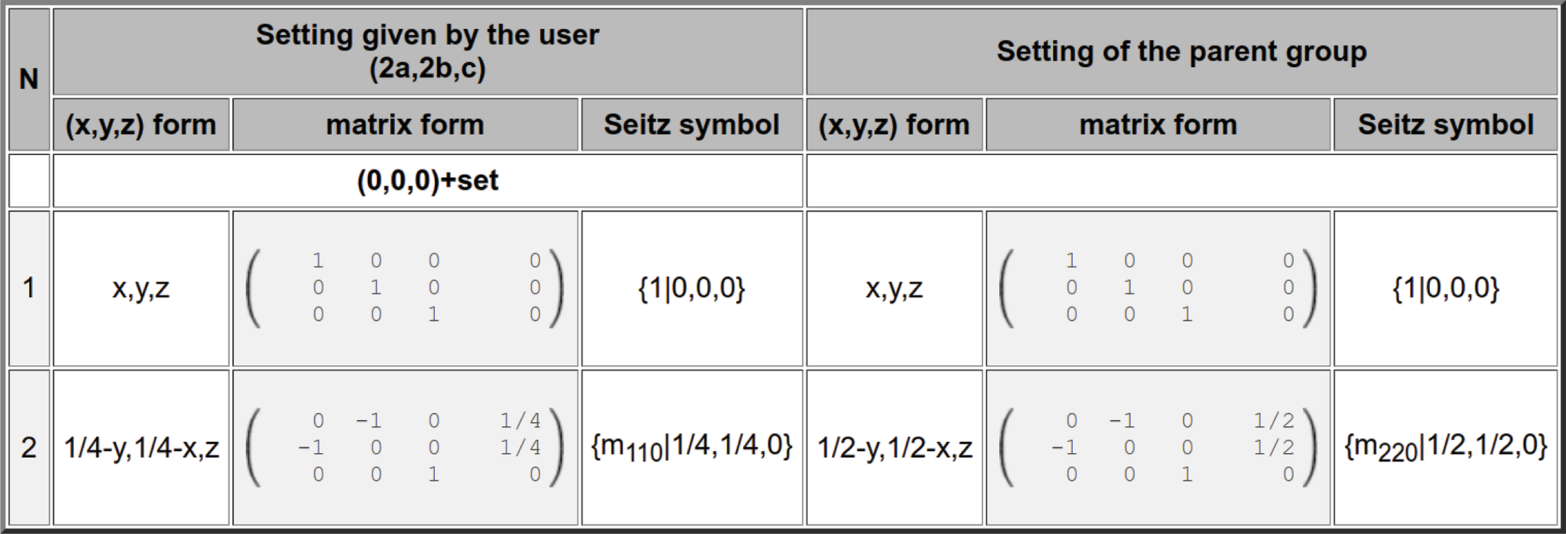
Symmetry operations (or general positions) of the subgroup with space-group type *Cm* and listed as 3.1 in the first row of Fig. 3, as obtained when clicking in the column ‘Symmetry operations’ (‘Matrix form’). Only one operation is listed, as a representative, for each set of operations differing by lattice translations of the subgroup. In this simple case the list is reduced to two operations. They are described in different formats, including the Seitz notation, and are given both in the basis generating the lattice of the subgroup (left-hand columns) and in the parent unit-cell basis (right-hand columns). In this latter case they can be identified as operations of the parent space group in its standard setting.

The output shown in Fig. 3 ▸, similar to that in Fig. 1 ▸, includes some options. In the fifth column (‘Symmetry operations’), there are two options that allow viewing the set of symmetry operations forming each of the listed subgroups, either in plain text format or in matrix form. Fig. 4 ▸ shows as an example the list of operations obtained for the first subgroup in Fig. 3 ▸ when the ‘Matrix form’ is selected.

**Figure 5:**
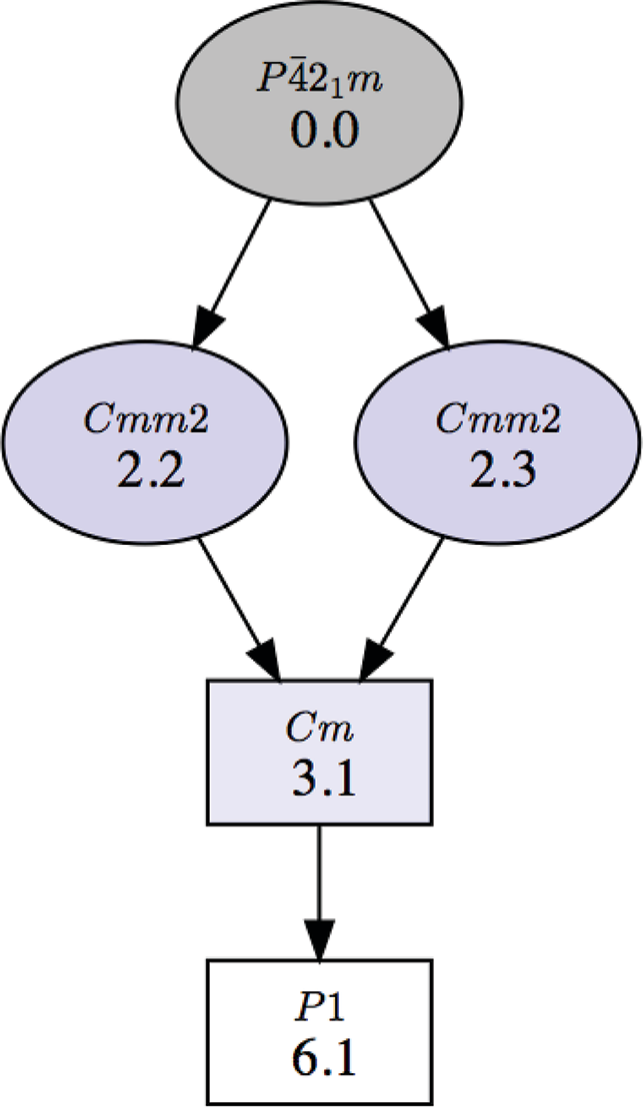
A group–subgroup graph showing the supergroups and subgroups of one of the *Cm* subgroups of 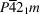 (No. 113), namely the one listed as 3.1 in Fig. 3. The numerical labels are those identifying the subgroups in the listings provided for each conjugacy class.

**Figure 6:**
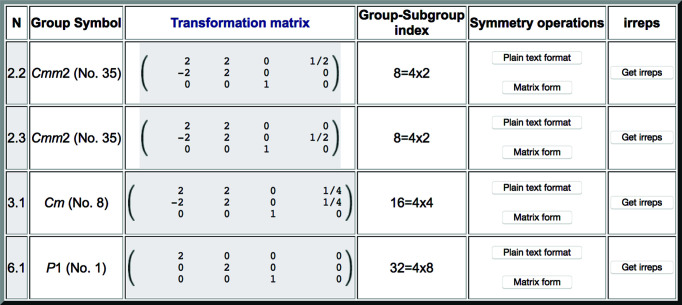
A list of the subgroups appearing in the graph of Fig. 5, as obtained when clicking on the button ‘List of subgroups’ for the subgroup indexed 3.1 in the output shown in Fig. 3.

Once a conjugacy class is listed, the group–subgroup relationship of each specific subgroup within the class, including both subgroups and supergroups, can be obtained using the optional buttons in the column entitled ‘Set of subgroups’ (Fig. 3 ▸). Figs. 5 ▸ and 6 ▸ show how this information is given for the case of the subgroup numbered 3.1 in Fig. 3 ▸. The group–subgroup hierarchy is depicted graphically by clicking on ‘Graph of subgroups’ (Fig. 5 ▸). This subgroup has two distinct supergroups of type *Cmm*2. Full information on the subgroups present in the graph (including their unambiguous definition) is provided when clicking on ‘List of subgroups’ (Fig. 6 ▸). As in the previous listing of Fig. 3 ▸, the output includes additional options for further information.

### 2.3. Irreducible representations of the parent space group compatible with each subgroup

The button ‘Get irreps’, which is generally available for each subgroup in the listings provided by the program (see Figs. 1 ▸, 3 ▸ and 6 ▸), is a direct link to the program *Get_irreps*, also available as a standalone online program in the BCS. By calling this program for one particular subgroup one gets all the irreducible representations (irreps) of the parent group which are compatible with the chosen subgroup (details of the calculations and mathematical definitions of the terms used herein can be found in Appendix *A*[App appa]). These are the irreps which can characterize the degrees of freedom that, in accordance with the Von Neumann principle, are symmetry allowed, and therefore they are necessarily set free in a distorted structure with its symmetry described by this subgroup. The program provides not only the irreps but also the subspace or direction required within the irrep space and the *isotropy subgroup* (or *epikernel*) associated with this irrep and direction. In the case of complex irreps, the program considers their combination into physically (real) irreducible representations.

The irrep labels used by the program are those of the irrep tabulations available in the BCS and in the *ISOTROPY Software Suite*. This is the CDML notation [Cracknell, Davies, Miller and Love; Cracknell (1979 ▸)] which is used by all programs in these two web facilities. In the case of physically irreducible representations, the irrep labels of the two irreps being combined are put together to form a single label.

**Figure 7:**
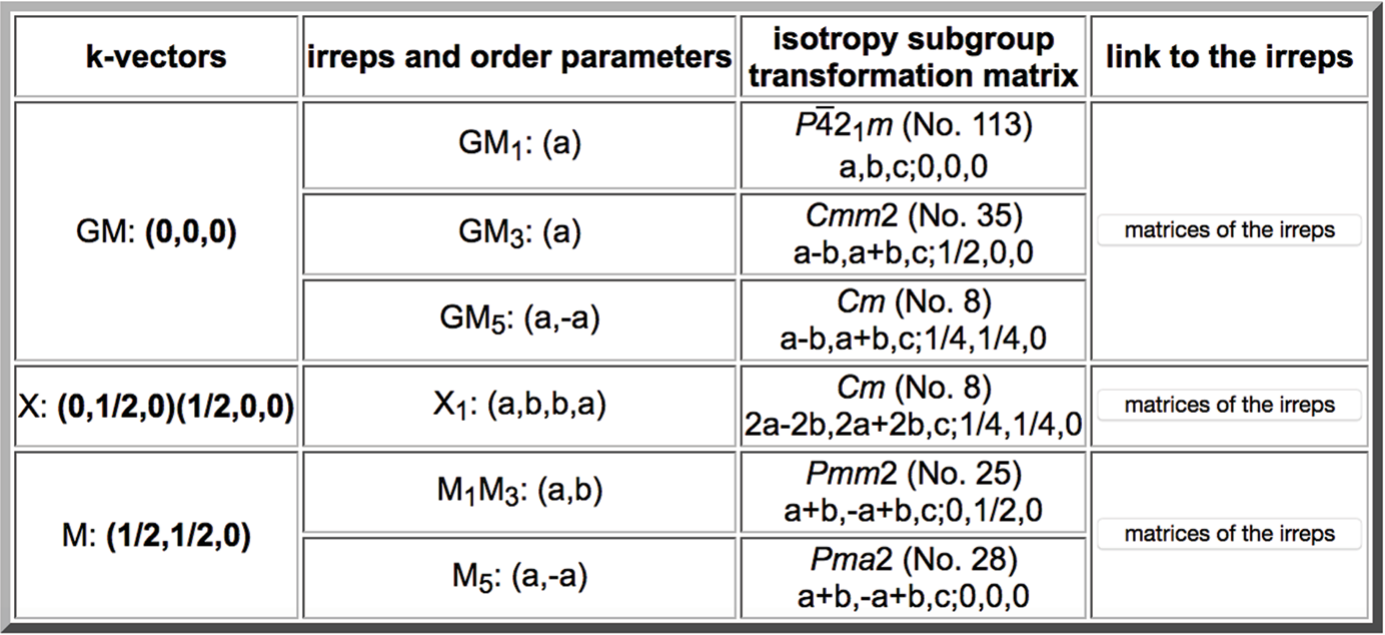
Irreducible representations (irreps) of the space group 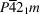 (No. 113) which are compatible with its subgroup of type *Cm* (2**a** − 2**b**, 2**a** + 2**b**, **c**; 1/4, 1/4, 0), listed as 3.1 in Fig. 3. This is the output obtained by clicking on the button ‘Get irreps’. The output lists the wavevectors involved for each irrep (in bold), the irrep label and the required direction within the irrep space. For each irrep, the isotropy subgroup or epikernel is also indicated. See the text for more details.

Fig. 7 ▸ shows the output obtained by calling *Get_irreps* in the case of the subgroup of type *Cm* numbered 3.1 in Fig. 3 ▸. The actual irrep matrices which are considered for the description of the relevant irreps can be consulted by clicking on the corresponding button ‘matrices of the irreps’ in the last column of the table. This is a direct link to the database *REPRESENTATIONS SG*, also available in the BCS, where the matrix form that is being used for the irreps can be retrieved. Although the CDML irrep labels are the same as those in the *ISOTROPY Software Suite*, the specific matrix form of the irreps considered in *REPRESENTATIONS SG* may be different. Therefore it is important to stress that the order parameter direction in the second column of the table in Fig. 7 ▸, which depends on the matrix choice for the irrep, is not necessarily the same as the one that may be obtained using the programs of the *ISOTROPY Software Suite*.

In the third column of Fig. 7 ▸ one can consult the isotropy subgroup associated with each of the listed compatible irreps. These *irrep isotropy subgroups* of the parent space group, also specified by a space-group type and a transformation (**P**, **p**) to its standard setting (given in a short-hand notation), are the symmetries that would only result from the presence of a distortion in the structure, transforming according to the corresponding irrep (restricted to the indicated subspace). By definition, all these subgroups must be supergroups of the actual subgroup being analysed, or coincide with it. In the latter case this symmetry break can be the result of a phase transition fulfilling the Landau theory condition (Landau & Lifshitz, 2013 ▸; Cowley, 1980 ▸) of a single irrep describing the transformation properties of its order parameter. Distorted structures very often comply with this Landau assumption, and therefore subgroups that can be reached by the onset of a single irrep and appear in their *Get_irreps* output as the isotropy subgroup of one of the irreps are more probable. We shall see in Section 3 that this condition can be applied as a filter. The list of compatible irreps and respective isotropy subgroups for the subgroup *Cm* (2**a** − 2**b**, 2**a** + 2**b**, **c**; 1/4, 1/4, 0) shown in Fig. 7 ▸ includes this subgroup itself for the four-dimensional irrep labelled X_1_ (or X1 – due to format limitations some output pages do not show the numbers in the irrep labels as subscripts but as ordinary fonts). Hence, this subgroup satisfies the Landau condition, as it can be reached by the presence of a distortion according to this single irrep X_1_, restricted within a two-dimensional subspace. The bold characters of the two wavevectors (0, 1/2, 0) and (1/2, 0, 0) of the irrep star in Fig. 7 ▸ indicate that both of them are involved in the distortion.

**Figure 8:**
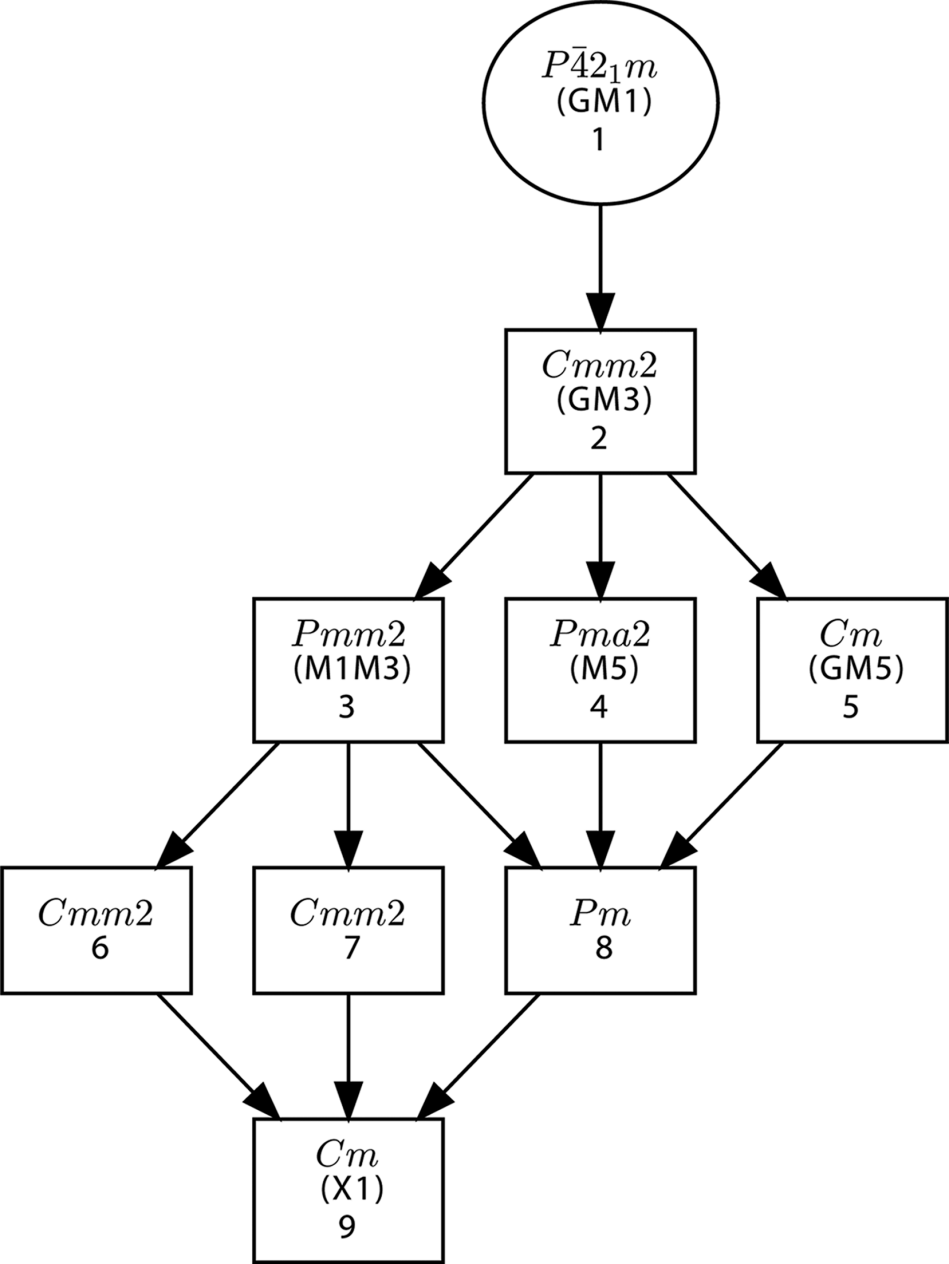
A group–subgroup graph showing all the intermediate subgroups between the specified subgroup of type *Cm* and the parent space group 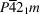 (No. 113), as obtained calling the program *Get_irreps*. The graph shows all intermediate subgroups, including those with lattices different from the one that is required for the subgroup *Cm*. In the case of those subgroups which are isotropy subgroups listed in Fig. 7, the corresponding irrep is also indicated. The numbers for each subgroup are those in the list provided by the program (not shown here), where the subgroups are fully defined.

The link to the program *Get_irreps* also permits the user to obtain a graphic representation of all the intermediate subgroups for the chosen subgroup, showing their group–subgroup hierarchy. In the case of the subgroups which are listed as isotropy subgroups, the graph also indicates the associated irrep. As an example, Fig. 8 ▸ depicts the graph that can be obtained as a complement to the output shown in Fig. 7 ▸. One can see that the end symmetry *Cm* can be reached with the single irrep X_1_.

The two subgroups of type *Cmm*2 in Fig. 8 ▸, numbered 6 and 7 (also listed in Fig. 6 ▸), are isotropy subgroups of the irrep X_1_, *i.e.* the same irrep for which the *Cm* subgroup is also an isotropy subgroup. This can be easily checked by calling *Get_irreps* for these two subgroups in the output shown in Fig. 6 ▸. But, in the case of these two higher subgroups, the direction within the irrep space is further restricted to a single free parameter. In such cases, the program only indicates for the irrep the isotropy subgroup corresponding to the most general distortion/direction allowed. In contrast, the intermediate subgroup of type *Pm* shown in Fig. 8 ▸ does not include any irrep label because it is not an isotropy subgroup for any irrep. The graph shows that this symmetry can only be attained through the combination of distortions according to at least two of the three irreps associated with its three immediate supergroups, namely M_1_M_3_, M_5_ and GM_5_.

### 2.4. Generation of CIF files of the parent structure under the selected subgroups

The rather comprehensive symmetry information provided by the program as explained above can be very useful when investigating a distorted or pseudo-symmetric structure. However, in many cases the first and most important problem is the actual determination of the distorted or pseudo-symmetric structure, either using diffraction data or through energy minimization in DFT calculations. To facilitate a straightforward use of the program when dealing with this type of problem, the list of symmetries, as in the example in Fig. 1 ▸, includes in the last column an option for each listed subgroup which introduces an automatic link to another tool of the BCS, namely *TRANSTRU*. If a CIF file of the parent structure is then uploaded, this option permits the automatic generation of a set of CIF files, one for each of the selected subgroups, where the parent structure is described under the subgroup symmetry in the standard setting of its space group type. The CIF files can then be used in refinements using diffraction data or in DFT energy minimizations, constrained to these alternative symmetries. Note that for monoclinic and triclinic symmetries the standard unit cell that *SUBGROUPS* may have chosen can be quite inappropriate, depending on the metrics of the parent lattice. It is then convenient to transform the CIF file to a description with a more adequate unit cell. *TRANSTRU* as a standalone program can also be used for this purpose. One just needs to introduce the same group for the group–subgroup pair, and the desired change in unit cell.

From a practical point of view, once the list of possible subgroups/symmetries is obtained in the first step explained in Section 2.1, and after applying, if necessary, some of the available filters (see Section 3), the user can skip all the optional detailed information about the subgroups and go directly to this last option to generate appropriate CIF files for the desired symmetries.

## 3. Filters

In order to reduce the number of potential symmetries generated by the program, different filters can be applied based on different criteria. These filters can be introduced on the first input page and serve to narrow down the enumeration process.

We stress that these filters are applied after the program obtains the full list of subgroups, which is done mathematically without any filter. This means that the application of any of the filters does not reduce the computing time. The program in fact may fail in cases where the number of possible unfiltered subgroups is extremely large, requiring a very long computing time, even if the filtered set were small. As the complexity of the branches belonging to the group–candidate-subgroup trees increases exponentially, and since each of these chains is handled separately proceeding through maximal subgroups all the way down to *P*1, a higher subgroup index might result in a long waiting time.

The filters that can be applied can be divided into different categories as follows.

### 3.1. Maximal subgroups

The simplest filters are those that limit the lowest symmetry to be considered. Without them the program lists all subgroups up to the lowest possible one. Alternatively, the list can be limited to the maximal subgroups, *i.e.* those subgroups for which no intermediate supergroup exists among those subgroups calculated by the program. Subgroups can also be limited to those being polar, non-polar, centrosymmetric or non-centrosymmetric, *etc*.

### 3.2. Displacive distortions

Another important filter exists for structures having very few independent atoms on special positions. In these cases, some of the subgroups mathematically calculated by the program cannot be attained by displacive distortions, *i.e.* by any kind of correlated atomic displacements. The reason is that, if all the atoms occupy special positions, a feasible subgroup must necessarily increase the number of free parameters necessary to define their positions with respect to all its immediate supergroups, otherwise this symmetry can never be attained by the displacements of these atoms because one of the supergroups with the same number of free parameters would be realized (see Appendix *A*2). The occupied Wyckoff positions can be specified and the program then drops from the list all these ‘impossible’ symmetries, while the subgroups only attainable by some lattice strain, if existing, can be included or excluded.

**Figure 9:**
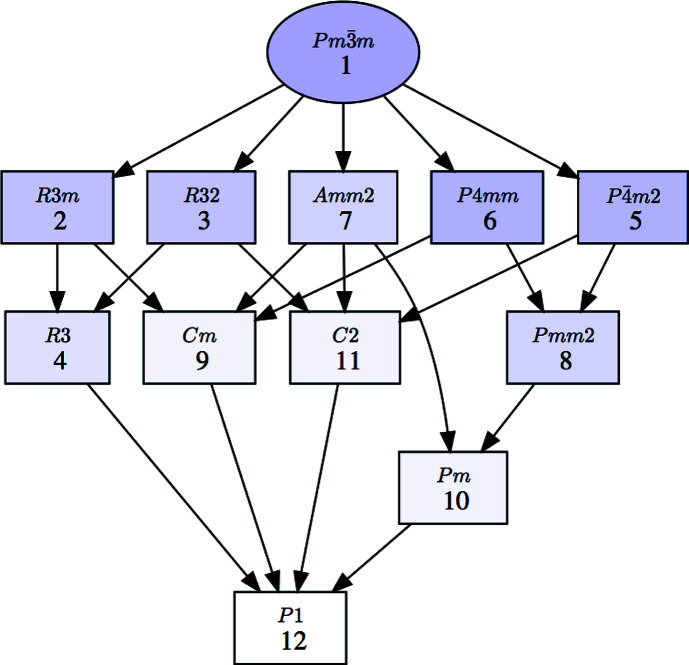
A group–subgroup graph showing all the possible subgroup symmetries that can have a distorted perovskite structure, *i.e.* a structure with parent space group 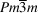 (No. 221) and occupied Wyckoff positions 1*a*, 1*b* and 3*d* (or 3*c*), if the distortion is of displacive type and the lattice is maintained. Symmetries only attainable by a lattice strain are not included.

As an example let us consider a parent structure with space group 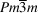 (No. 221) and three symmetry-independent atoms on the Wyckoff positions 1*a*, 1*b* and 3*d*, *i.e.* the ideal prototype structure of a perovskite. If we are interested in possible distorted perovskites which keep the parent lattice, and therefore we introduce as ‘supercell’ the same parent unit cell, the number of possible distinct symmetries (conjugacy classes of subgroups) provided by *SUBGROUPS* without applying any filter is 33 (including 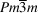 itself as a trivial case mathematically fulfilling the subgroup condition). If, however, the three mentioned Wyckoff positions are introduced as the only occupied ones, the list is then reduced to 19 subgroups. Optionally, the subgroups which are only attainable through lattice strains can also be excluded and the list is then reduced to 12 classes (always including the parent space group). There are therefore 11 space groups which can describe the symmetry of a distorted perovskite resulting from a displacive distortion that (approximately) keeps the parent lattice. Their group–subgroup hierarchy is shown in Fig. 9 ▸ (the ordering does not indicate the index levels but has been arranged with respect to maximal subgroup chains). We stress that this optional filter is intended to limit the possible symmetries to those caused by atomic displacements. Therefore it should not be applied if the distortion may include some type of order–disorder phenomenon, with the occupancy of some atomic sites varying between the parent and distorted structures.

### 3.3. Landau condition

**Figure 10:**
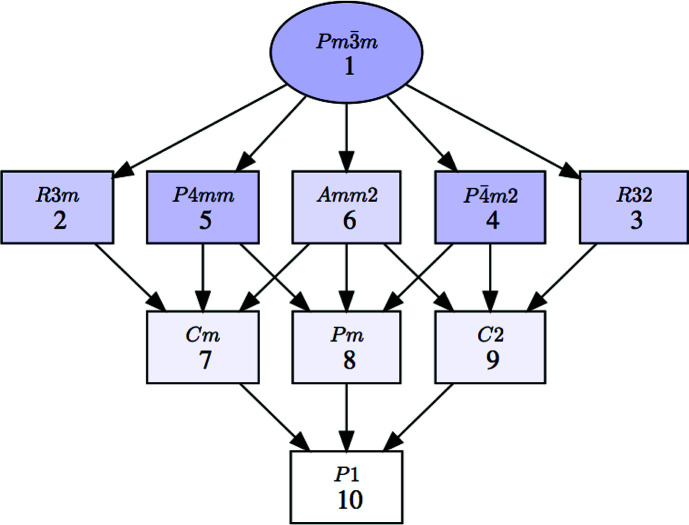
A group–subgroup graph showing all the possible subgroup symmetries that can have a displacive distorted perovskite structure as the result of a Landau-type phase transition with a single order parameter, with the lattice maintained.

The most important filter that the program provides is probably the one that restricts the enumeration of subgroups to those that can be attained with a Landau-type phase transition, *i.e.* to those subgroups corresponding to symmetry breaks which can be explained by the presence of a distortion transforming according to a single irreducible representation of the parent space group. This means that, following Landau theory (Landau & Lifshitz, 2013 ▸; Cowley, 1980 ▸), a single order parameter according to a single irrep can be introduced to describe a phase transition between the two symmetries. This filter can be fundamental to restricting a huge number of mathematically possible symmetries to just a few which can be considered most probable from a physical viewpoint. In the case of the example shown in Section 2 of a parent space group 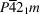, this filter is ineffective since all the subgroups listed in Fig. 1 ▸ fulfil the Landau condition, but in the second example considered in Section 3.2 of a parent space group 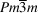 and with the lattice maintained, the number of symmetries reduces from 32 to 23. In the case of a perovskite-like structure and a displacive distortion, the 11 possible symmetries mentioned in Section 3.2 reduce to nine. Their group–subgroup hierarchy (see Fig. 10 ▸) shows that there are five which are maximal symmetries and these would be the first ones to explore.

### 3.4. Distortions according to one or several specific irreps

If the wavevector(s) option is used to introduce the lattice relation between parent and distorted structures, the enumeration of possible symmetries can be limited to those resulting from a distortion transforming according to one or more specific irreps associated with the input wavevector(s). The filtering to symmetries resulting from the simultaneous presence of more than one irrep is limited to a single wavevector or an irrep star of wavevectors.

**Figure 11:**
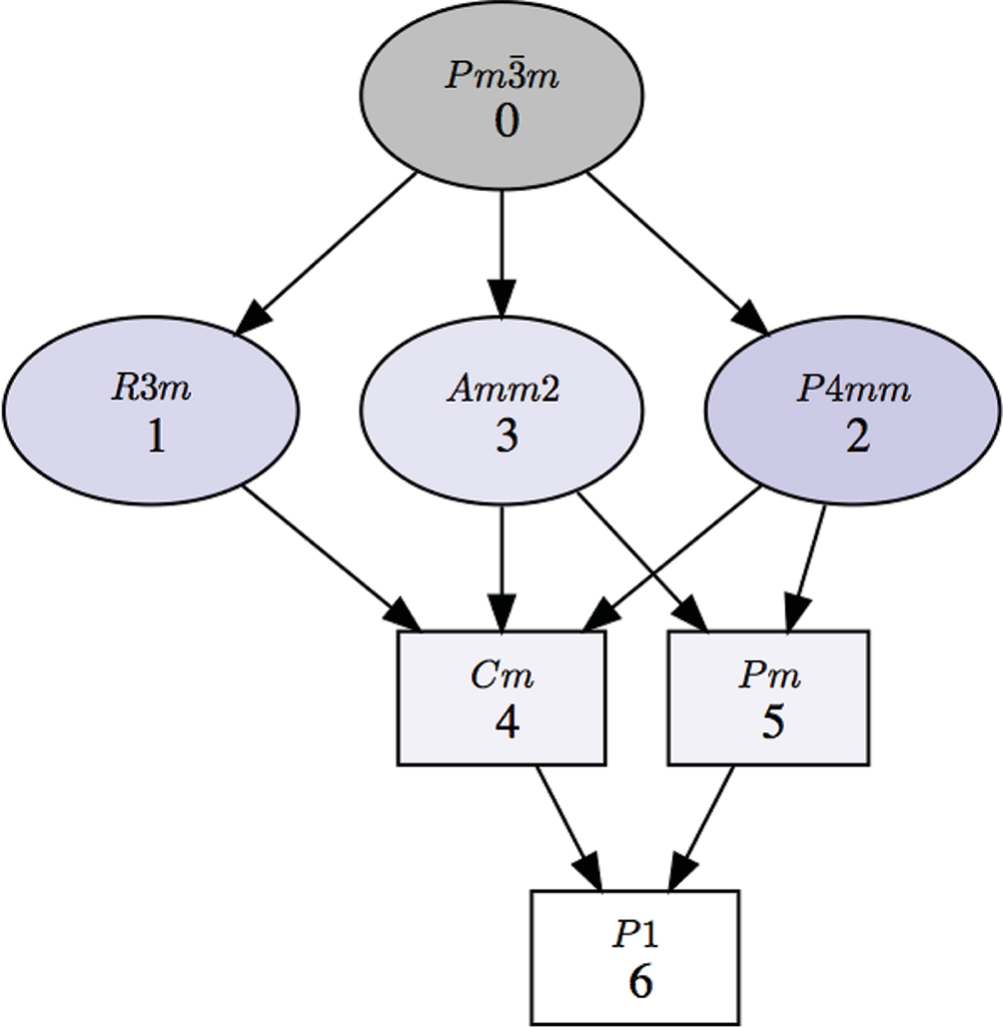
A group–subgroup graph showing all the possible subgroup symmetries which can have a distorted structure with parent space group 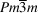 (No. 221) as the result of a distortion according to the polar irrep 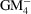 with **k** = (0, 0, 0). The different symmetries correspond to different order parameter directions within the three-dimensional irrep space.

Fig. 11 ▸ shows the graph obtained for the subgroups of the space group 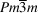, which the program enumerates if this filter is applied for the irrep 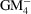 with **k** = (0, 0, 0). This irrep is the one associated with any kind of polar displacive distortion, and one recognizes in the figure all the space groups that have been observed in perovskite-like compounds exhibiting some proper ferroelectric phases due to a polar distortion.

## 4. Examples of application

These examples are all explained in more detail in the tutorial of the program, which is available on its webpage (https://journals.iucr.org/b/services/about.html).

### 4.1. Symmetry of the low-temperature phase of fullerene–cubane

Crystals that include molecules of both fullerene and cubane are known to crystallize at high temperatures according to the 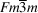 (No. 225) space group, with the disordered fullerenes centred on the site 4*a* (000) and the disordered cubane molecules on 4*b* (½½½). At low temperature, as these molecules become ordered, the system exhibits a phase transition into an orthorhombic phase. From powder diffraction experiments, the final low-temperature phase was reported to be a non-centred orthorhombic structure, with the parameters of its primitive unit cell satisfying the approximate relations *a* ≃ *b* ≃ *a*_c_/2^1/2^, while *c* ≃ 2*a*_c_, where *a*_c_ is the cell parameter of the cubic phase (Pekker *et al.*, 2005 ▸). However, the space group of this phase could not be determined and the structure remained unknown for several years (Bortel *et al.*, 2006 ▸). It took five years finally to identify the space group and determine the corresponding structure (Bortel *et al.*, 2011 ▸). Obviously, if the possible symmetry of this phase could have been restricted to a minimal set of space groups, there would have been a better chance of succeeding in the interpretation and analysis of its diffraction diagram when this structure was initially investigated. It is shown below that, using *SUBGROUPS*, the most probable space groups consistent with the observed cell parameters can be reduced to two. One of them is indeed the symmetry group of the structure that was finally determined in 2011.

The metrics of the reported primitive orthorhombic unit cell clearly indicate that its relation with the cubic cell of the non-distorted parent structure must be of the form **a**_s_ = **a**/2 − **b**/2, **b**_s_ = **a**/2 + **b**/2, **c**_s_ = 2**c**, where **a**, **b** and **c** define the conventional centred cubic unit cell of the parent structure. Introducing just the parent space group 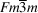 and this supercell (as primitive) in the first input page of the program, *SUBGROUPS* provides quite a long list of 99 possible subgroups (rigorously, a list of conjugacy classes, as explained above), which is consistent with the input supercell. Note that the program permits the user to define the supercell with some centring. Therefore, instead of the primitive supercell indicated above, one can introduce a *C*-centred supercell **a**_s_ = **a**, **b**_s_ = **b**, **c**_s_ = 2**c** and the result is just the same, as both supercells define the same lattice. The cubic symmetry of the parent space group also means that the interchange of cell parameters in the supercell definition will result in the same list of conjugacy classes of subgroups.

**Figure 12:**
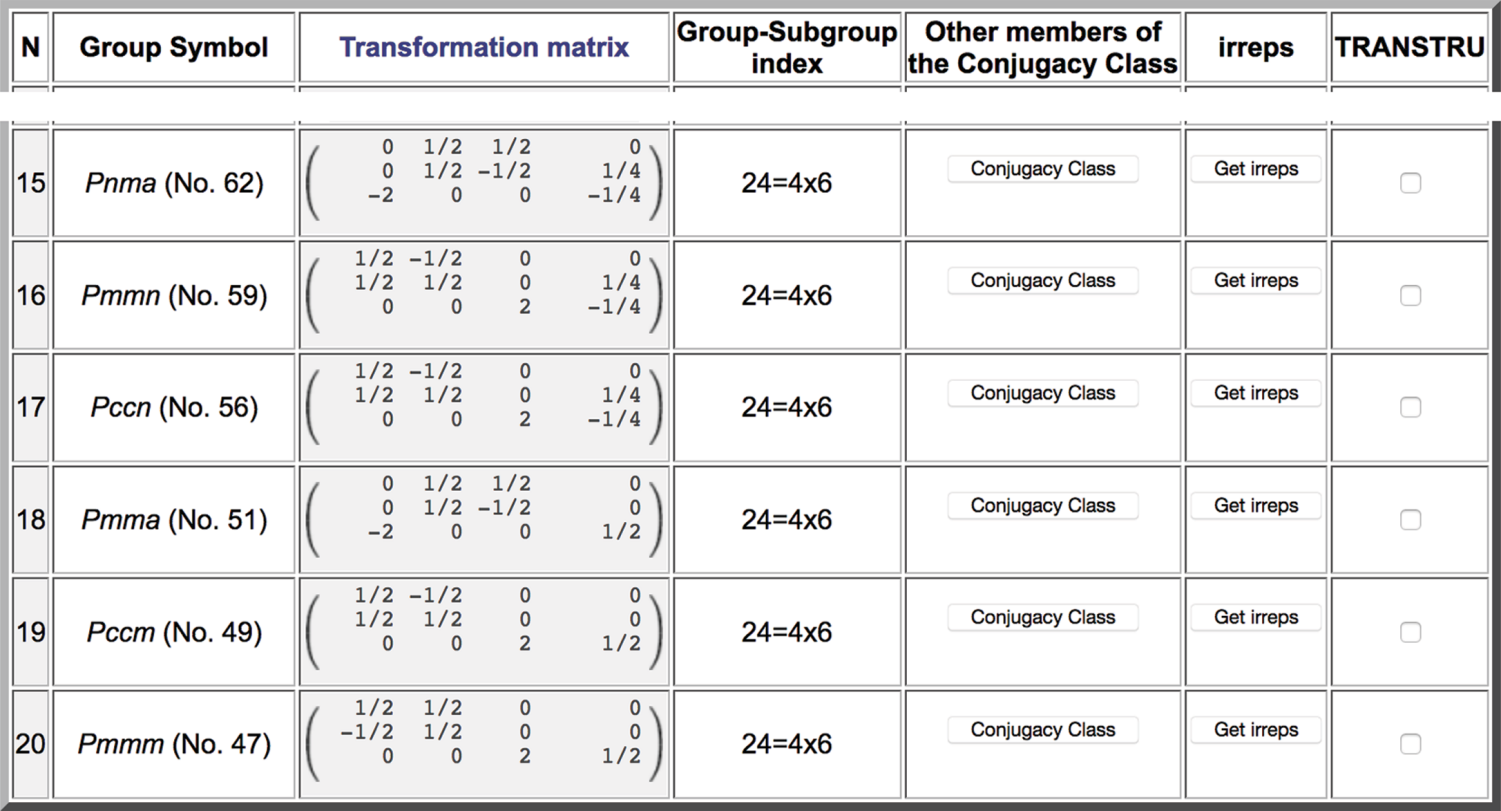
A fragment of the list of subgroups obtained with *SUBGROUPS*, showing only the possible non-centred space groups with point group *mmm* for a structure resulting from the distortion of a cubic structure with space group 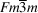 (No. 225), and having as primitive unit cell a supercell (**a**/2 − **b**/2, **a**/2 + **b**/2, 2**c**) or equivalent.

The list of possible symmetries is extremely large because by default the program lists up to the lowest possible symmetry with space group *P*1. But as the lattice is reported to be orthorhombic, we can include the filter that only higher-symmetry subgroups up to the orthorhombic crystal family should be listed. The list is then reduced to 62 subgroups, but most of them can still be discarded as the list includes all subgroups belonging to crystal systems higher than the orthorhombic. Furthermore, among the orthorhombic space groups are also listed those not belonging to the holohedry, with point groups 222 and *mm*2. As maximal symmetries are usually realized, we are going to assume that the relevant point-group symmetry is the maximal one within the ortho­rhombic class, *i.e.* the holohedry *mmm* (if this assumption were to turn out to be unsuccessful, one could then always proceed similarly with the other two possible orthorhombic point groups). By applying the filter ‘Lowest point group to consider: *mmm*’, the list is then reduced to 20 subgroups. This list includes all subgroups with point group *mmm* or higher. It therefore includes tetragonal groups, which can be directly discarded, and also *C*-centred orthorhombic groups, which can also be excluded for the following reason: the unit cell of the investigated structure is known to be primitive with the ortho­rhombic axes along the oblique basis directions of the supercell, while the *C*-centred orthorhombic groups have their orthorhombic symmetry axes along the parent cubic **a**, **b**, **c** directions. Note that the program correctly includes these *C*-centred orthorhombic subgroups in the list, because they have as primitive unit cell the one that has just been introduced. The possible symmetries are then reduced to the six shown in Fig. 12 ▸.

**Figure 13:**
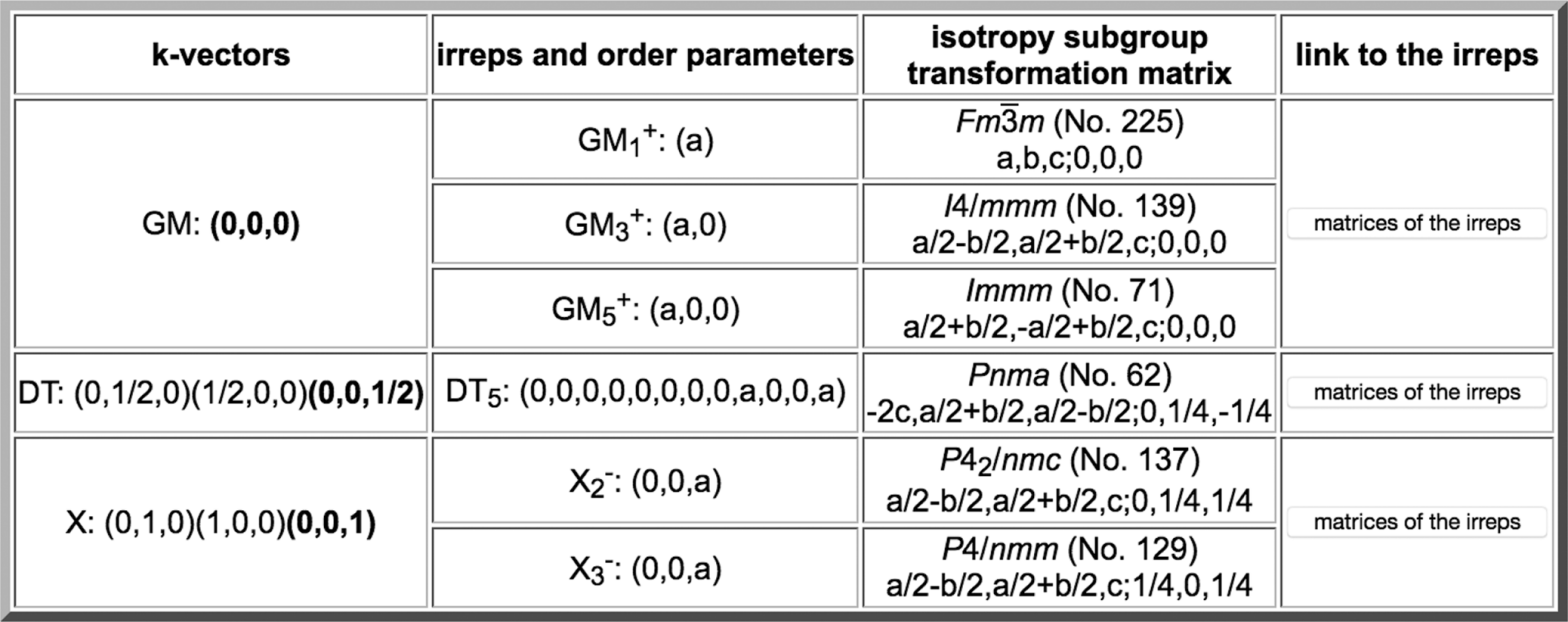
The output of *SUBGROUPS* when calling *Get_irreps* for the subgroup of 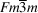 of type *Pnma* (No. 62) indicated in Fig. 12. One can see that this *Pnma* subgroup is in fact among the listed compatible irrep isotropy subgroups, and therefore this symmetry can be attained by an order parameter associated with the corresponding irrep, namely DT_5_. The wavevector (0, 0, ½), shown in bold, within the set of wavevectors of the irrep (irrep star) is the only one involved in the irrep distortion necessary for this symmetry break.

By clicking on *Get irreps* for the first subgroup of type *Pnma* (No. 62) listed in Fig. 12 ▸, we obtain the irreps compatible with this symmetry break. One can see in the output shown in Fig. 13 ▸ that this subgroup itself is an isotropy subgroup (epikernel) for the irrep DT_5_ with wavevector (0, 0, 1/2). This implies that the symmetry break 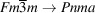 (−2**c**, **a**/2 + **b**/2, **a**/2 − **b**/2; 0, 1/4, −1/4) can be realized through a Landau-type phase transformation, with an order parameter having the transformation properties of a single irrep (within the specified direction).

If we apply the filter that limits the listed symmetries to those fulfilling the Landau condition, from the six possible orthorhombic symmetries shown in Fig. 12 ▸ only two survive, namely *Pnma* (No. 62) and *Pmma* (No. 51). These two symmetries should therefore have been the first ones to explore for the investigated phase. Of course, the other four orthorhombic symmetries could also be possible, but they necessarily require the action of more than one irrep, *i.e.* the presence of at least two order parameters. The symmetry of the structure, which was finally reported by Bortel *et al.* (2011 ▸), is in fact given by the *Pnma* subgroup mentioned above. Note that in this case the filter indicating the occupied Wyckoff positions is not applicable, because the transition is not displacive but of order–disorder type, and the occupied Wyckoff positions refer to the centres of disordered molecules.

### 4.2. Possible distorted structures resulting from the onset of a degenerate unstable mode

High-symmetry structures often exhibit distorted phases associated with the instability of ‘rigid-unit modes’ (RUMs) [see Campbell *et al.* (2021 ▸), and references therein], which involve correlated rotations of frameworks of ionic polyhedral units sharing vertices while maintaining their rigidity in a first approximation. These RUMs are usually associated with multidimensional irreps and are therefore degenerate in the high-symmetry parent structure. Different combinations of these degenerate RUMs can then give place to different distorted phases with different symmetries, all described by subgroups of the parent space group. The perovskite structure is an example of this situation.

A typical RUM, which is soft or fully unstable in many perovskites, is for instance the one that becomes frozen in SrTiO_3_ below 105 K and results in a phase with space group *I*4/*mcm* (No. 140). If we choose the origin in the cubic perovskite such that the Sr atom lies at the origin, this tetragonal symmetry implies the following symmetry break,
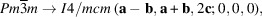
where we have chosen, among the three equivalent conjugate subgroups, the one with the orientation of the tetragonal axis along the cubic *c* axis. The indicated null origin shift of the transformation to the standard setting corresponds to the origin choice in the parent structure mentioned above. In this example we will first use *SUBGROUPS* to identify the irrep associated with the RUM active in the distorted phase of SrTiO_3_, showing that it is threefold degenerate in the cubic phase. The program will then be used to explore all the possible symmetries that can occur in a perovskite as the result of a distortion due to this kind of unstable RUM.

**Figure 14:**
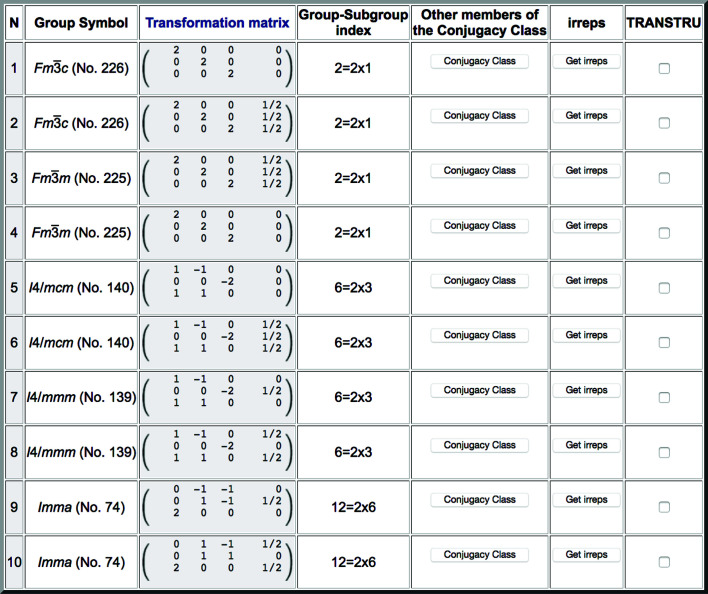
Possible maximal symmetries for a distorted structure with parent space group 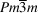 (No. 221) having an *I*-centred supercell (**a** – **b**, **a** + **b**, 2**c**) with respect to the cubic unit cell. The same results are obtained if an *F*-centred supercell (2**a**, 2**b**, 2**c**) or a primitive supercell (**a** + **c**, **b** + **c**, 2**c**) is introduced, as they define the same sublattice for the distorted structure.

If we restrict *SUBGROUPS* to all the maximal subgroups (conjugacy classes) with an *I*-centred supercell of the type observed in SrTiO_3_, the obtained list is shown in Fig. 14 ▸. Among the ten listed possible maximal symmetries, the conjugacy class corresponding to the symmetry of the low-temperature phase of SrTiO_3_ is present, numbered 5 in the list. The subgroup chosen as representative by the program for this class is not the one we defined above, but inspecting the full conjugacy class one can check that the latter is formed by three subgroups with the three possible relative orientations of the tetragonal axis. There is another conjugacy class (number 6) with subgroups of type *I*4/*mcm*, but they are distinguished by the position of the standard origin with respect to that of the parent structure. Note that, if we shift the origin of the parent cubic structure (1/2, 1/2, 1/2) such that it is the Ti atom that lies at the origin, we would then have to consider the conjugacy class numbered 6 in Fig. 14 ▸, instead of the fifth one, as the one relevant for the symmetry of the distorted phase of SrTiO_3_.

**Figure 15:**
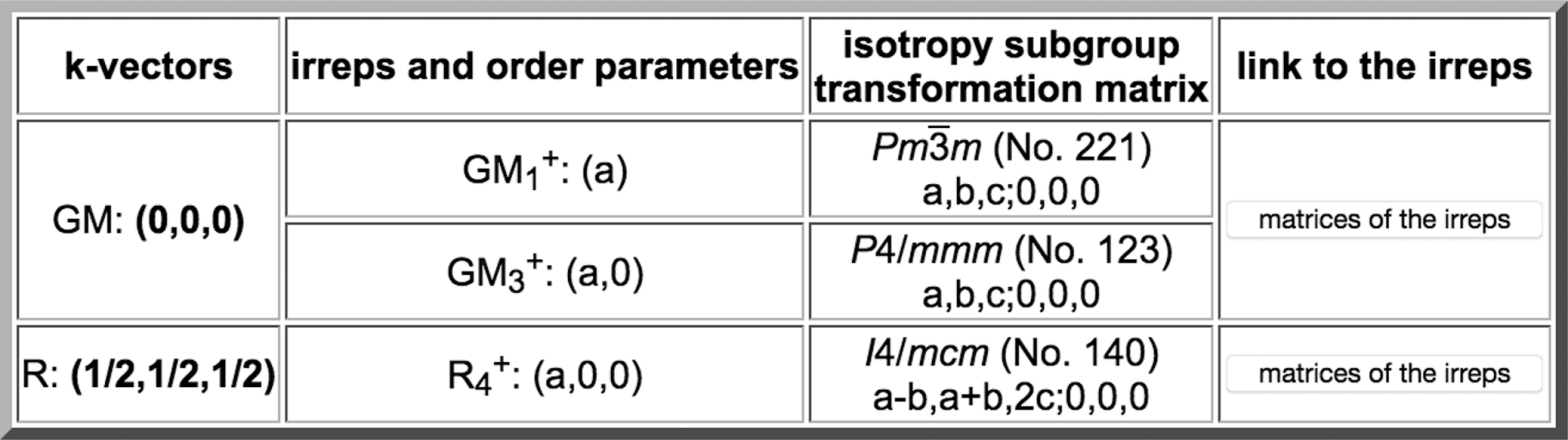
The result of calling the program *Get_irreps* within the conjugacy class numbered 5 in Fig. 14 for the subgroup *I*4/*mcm* (**a** − **b**, **a** + **b**, 2**c**; 0, 0, 0). One can see that the irrep 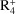 with wavevector (½, ½, ½) (point *R* in the Brillouin zone) is responsible for this symmetry break.

Using the direct link to the program *Get_irreps* for the relevant subgroup within the conjugacy class numbered 5 in Fig. 14 ▸, one obtains the set of irreps compatible with this subgroup listed in Fig. 15 ▸. The irrep 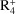 can be identified in this output as the one directly yielding the observed symmetry, and therefore it should be the irrep that describes the transformation properties of the RUM responsible for the distorted phase of SrTiO_3_. One can see in Fig. 15 ▸ that this irrep is three dimensional and the observed symmetry requires a special direction (*a*, 0, 0) within the irrep space. This means that there are three modes, degenerate in the cubic structure, and an arbitrary combination of them will produce a general 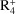 distortion, represented by an arbitrary direction (*a*, *b*, *c*) in the 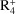 irrep space. The only symmetry operations that will be preserved by such a general 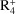 distortion are those with which the irrep 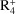 associates the 3 × 3 identity matrix, and which therefore remain invariant under any 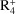 distortion. These operations form a subgroup of the parent space group, the kernel of the irrep, which is the minimal symmetry for an 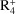 distortion. For special combinations of the three modes, *i.e.* special directions of the three-dimensional 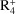 order parameter, the preserved symmetry can be higher, corresponding to supergroups of the mentioned kernel, *i.e.* the irrep epikernels or irrep isotropy subgroups already mentioned. One example is the (*a*, 0, 0) direction, corresponding to the presence of only one of the basis modes and resulting in the subgroup of type *I*4/*mcm*, as shown in Fig. 15 ▸. But there are other possible special directions or special combinations of the three RUMs, yielding other isotropy subgroups. As shown below, *SUBGROUPS* can provide detailed information on all of them and supply CIF files of the parent structure under each of these symmetries.

**Figure 16:**
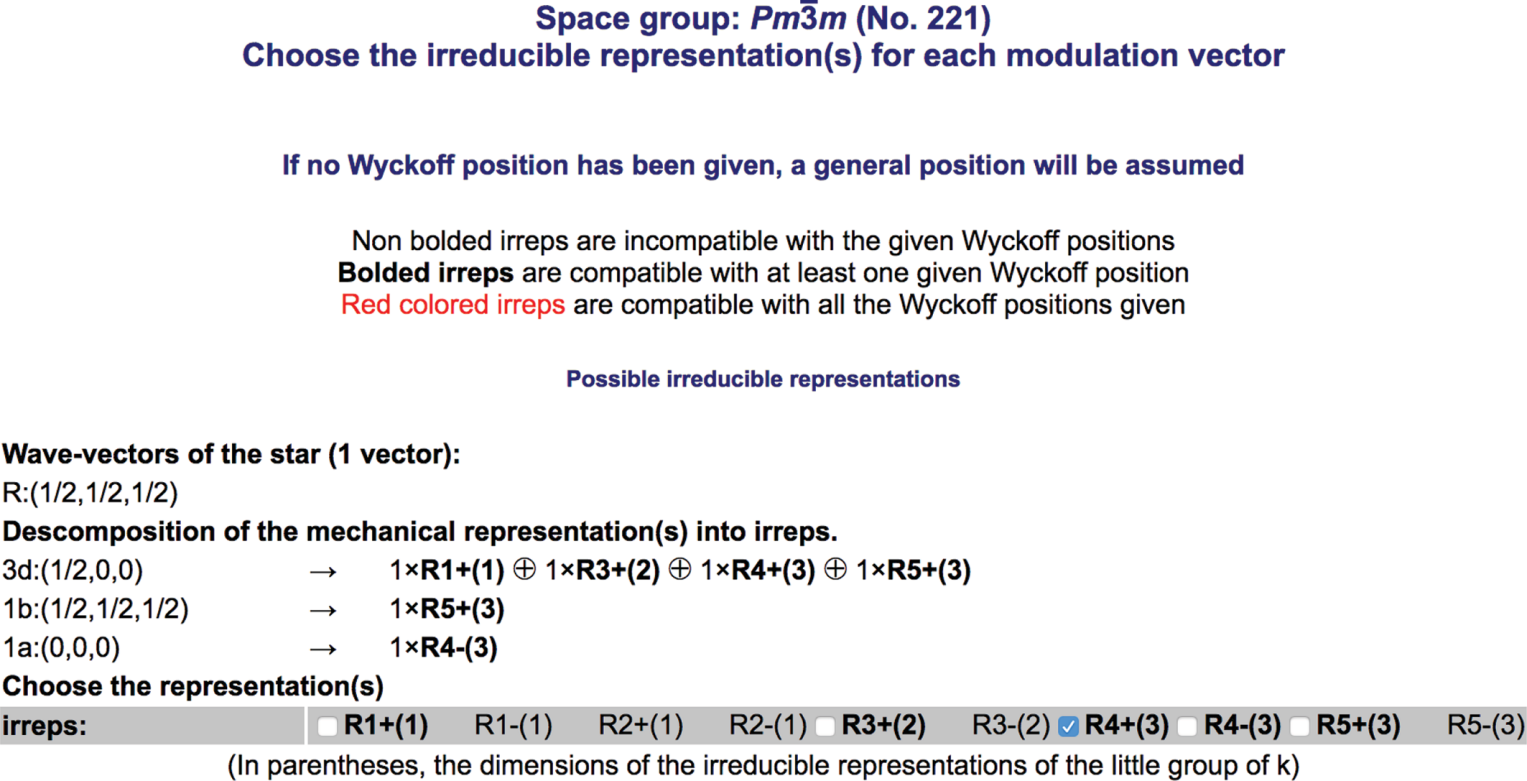
A partial view of the output and input pages of *SUBGROUPS* when ‘Representations’ is clicked in the input page of *SUBGROUPS* after introducing the wavevector and the occupied Wyckoff positions. The irrep decomposition of the space of displacive distortions (mechanical representation) for the wavevector *R* (½, ½, ½) is shown. One can then choose one or more of the listed possible irreps and reduce the listing of possible subgroups to those resulting from the onset of order parameter(s) associated with the chosen irrep(s).

**Figure 17:**
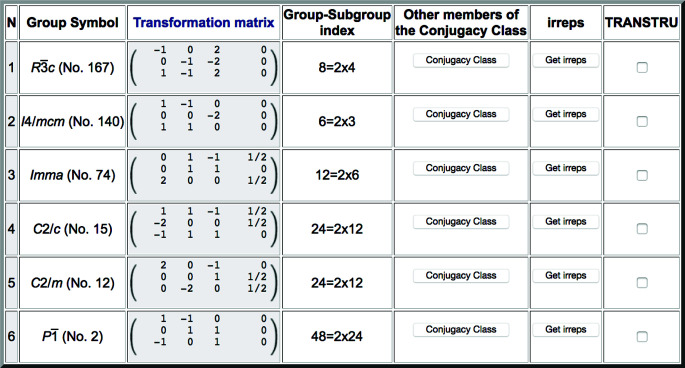
Possible space-group symmetries for a distorted structure derived from a 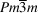 (No. 221) parent structure, as the result of a distortion according to the 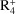 irrep.

We can now use the alternative input option and introduce the modulation wavevector (½, ½, ½) of the distorted structure instead of its supercell. This permits the use of the filter restricting the subgroups to specific irreps. After indicating the occupied Wyckoff positions as well, we obtain the decomposition into irreps of the representation describing the transformation properties of the possible displacive distortions (the mechanical representation) (Fig. 16 ▸). One can then see that only four of the ten possible irreps at the point *R* are relevant for a perovskite structure, and among them, as expected, the 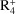 irrep is present and involves only the oxygen atoms (occupying position 3*d*). One can choose one or several of these irreps and see the possible symmetries resulting from the presence of distortions associated with them. We are, however, interested in those resulting from the freezing of some combination of the RUMs according to the 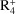 irrep. The output for this choice is shown in Fig. 17 ▸. The possible subgroups (strictly, conjugacy classes) are therefore six, with 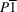 being the minimal symmetry.

**Figure 18:**
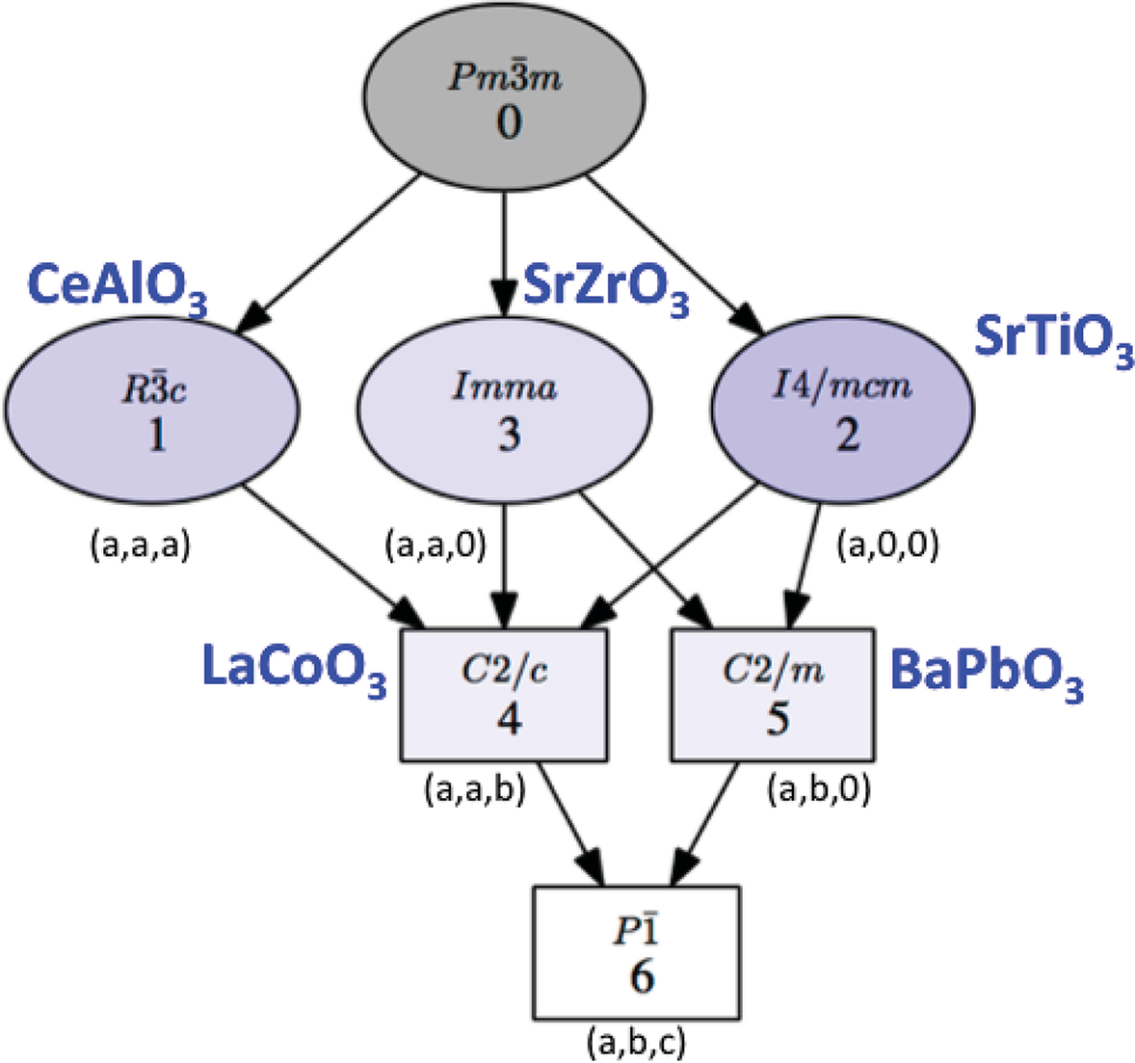
A graph showing the possible space-group symmetries for a distorted structure derived from a 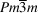 (No. 221) parent structure, as the result of a distortion according to the 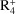 irrep. The graph provided by *SUBGROUPS* has been complemented with an indication of an appropriate direction of the order parameter for each symmetry. Examples of perovskite-related materials where these symmetries are realized are also indicated.

The graph depicting the group–subgroup hierarchy of the six possible isotropy subgroups of the 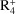 irrep is shown in Fig. 18 ▸. The graph provided by the program has been complemented with an indication of the order parameter directions corresponding to each symmetry. The 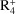 distortion modes, being RUMs in the perovskite structure, are expected to cost little energy, or even to be unstable and become frozen. One therefore expects that the symmetries shown in Figs. 17 ▸ and 18 ▸ are realized in some perovskite-like materials. This is indeed the case, and we have also indicated in Fig. 18 ▸ for each of the possible space groups (except for the minimal kernel) a specific compound where this particular symmetry has been observed.

## 5. Conclusion

Through the systematic application of group theory, the program *SUBGROUPS* can greatly reduce the number of potential candidates for the symmetry of a distorted phase. The program, in its essence, requires a few parameters, such as the symmetry group of the parent structure and the relation of the lattice of the distorted or pseudo-symmetric structure to that of the parent one. Usage of additional options enables further filtering, while it is also possible to conduct the analysis via inputting the distortion modulation vector(s).

In its output, we have aimed to present as many details as possible, including, but not limited to, the identification of active irreps and classifications by conjugacy classes and subgroup indices, along with graphs complementing the results presented in the lists and tables. If a CIF file of a structure described in the parent space group is uploaded, the program generates CIF files where the reported structure is represented under a set of selected subgroups. These CIF files of possible candidates of the distorted structure can be used for further refinement against diffraction data or for energy minimization in DFT calculations.

The use of the program is straightforward and does not require a deep knowledge of group theory, as the intermediate calculations are done automatically by making internal calls to other tools of the BCS, and the user is guided to those additional tools should further details be desired.

Different applications of the program have been outlined using two examples of known materials. Some additional detailed examples are included in the tutorial which can be downloaded from the main page of the program (https://journals.iucr.org/b/services/about.html).

## Footnotes

1 For space groups with more than one description in *IT*A, the following settings are chosen as standard in the BCS: *unique axis b* setting, *cell choice 1* for monoclinic space groups, *hexagonal axes* setting for rhombohedral space groups, and *origin choice 2* (origin at −1) for centrosymmetric space groups with more than one conventional choice of the origin.

## Appendix A Algorithms used by *SUBGROUPS*

Given the space group  of the parent structure and a supercell, independently of the filters introduced in the input, *SUBGROUPS* first calculates the whole set of subgroups compatible with the supercell. If the user has introduced a set of modulation wavevectors instead of the basis vectors of the supercell, the program calculates the supercell determined by those wavevectors and uses this supercell in the calculation of the subgroups. In this appendix we first describe the general algorithm to calculate all the subgroups without filters and then we focus on the most relevant filters used by the program.

### A1. Determination of the subgroups for the default (minimal) input: space group and supercell

At the start, we have a set of symmetry operations {*R*^s^|**t**^s^} of  in the standard setting defined by the three basis vectors (**a**^s^, **b**^s^, **c**^s^). We define *R*^s^ as the 3 × 3 matrix representation of an operation of the point group  of , acting on the components of a point in space, and  are the three components of the translation also expressed in the basis (**a**^s^, **b**^s^, **c**^s^). The matrix *S* introduced by the user (or calculated from the set of wavevectors introduced by the user) relates a set of primitive basis vectors of the supercell (**a**, **b**, **c**) with (**a**^s^, **b**^s^, **c**^s^),

In the first step, the program transforms the symmetry operations of the parent space group into the basis of the supercell via the transformation

and makes a list of all operations (2) mod 1 in the translations.

In general, some point-group operations do not keep the supercell invariant and therefore cannot belong to any subgroup compatible with the given conditions. These operations can be easily identified because they have at least one non-integer component and are thus removed from the list. With the remaining point-group operations we construct a list of operations {*R*_*i*_|**t**_*j*_} with *i* = 1,…, *n*_*P*_ and *j* = 1,…, *i*_*k*_. *n*_*P*_ is the number of point-group operations compatible with the supercell which form a point group denoted  in the following. *i*_*k*_ (*klassengleich* index) is the number of cosets in the decomposition of the translation group of the parent space group with respect to the translation group defined by the supercell. We can further reduce the number of symmetry operations from the previous list of length *n*_*P*_ × *i*_*k*_: if *n* is the order of *R*, {*R*|**t**}^*n*^ = {*E*|**T**}, where **T** is some translation of the parent lattice. However, if **T** mod1 ≠ 0, the presence of {*R*|**t**} in a subgroup would imply the existence of a centring operation in the subgroup and, therefore, the resulting lattice would be different from the lattice assumed in the input. Once the final set {*R*_*i*_|**t**_*j*_} of allowed operations has been identified, the problem reduces to the search of all possible combinations of these operations that form a group.

*SUBGROUPS* calculates this list of subgroups ‘on the fly’ and therefore it is very important to try to reduce the number of operations in the calculations to make the program run as fast as possible. The algorithm or strategy used by *SUB­GROUPS* is as follows.

(i) It first identifies the point group  among the 32 crystallographic types of groups. This is almost immediate if we consider the list of *n*_*P*_ factors |*R*_*i*_| × *n*_*i*_, where |*R*_*i*_| = ±1 is the determinant of the matrix *R*_*i*_ and *n*_*i*_ is the order of the *R*_*i*_ operation. This list, together with the existence or non-existence of inversion symmetry, unequivocally identifies the point group among the 32 possible types of groups.

(ii) Using the previously tabulated group–subgroup trees for all of the 32 crystallographic types of point groups, we can thus match the group–subgroup tree of . We have also previously divided the groups into conjugacy classes and selected, for any non-trivial group, one of its maximal subgroups in the tree and the coset representatives of its decomposition with respect to the chosen maximal subgroup. We stress that, for crystallographic point groups, the number of coset representatives is either two or three (and one of them can be chosen as the identity *E*).

(iii) Making use of the information obtained in the previous step and with the aim of reducing the computation time, we further divide the point groups into two subsets:

*List A*. This contains the representatives  of the corresponding conjugacy classes (one group for each conjugacy class). For each point group in this list (except for the trivial one), we also identify a maximal subgroup  =  of  for some *j* and one or two coset representatives *R*_1_ (or *R*_1_ and *R*_2_ = ) in the decomposition with respect to the chosen maximal subgroup (we remove the identity from the list of coset representatives) such that

*List B*. The groups  in this list are those that belong to the conjugacy class of one of the point groups in list *A*. In this case we identify the point group  in *A* that belongs to the same conjugacy class to which  belongs and a symmetry operation {*R*|**t**} such that

(iv) Once the point groups have been classified, we start the calculation of the (space) subgroups from the lowest point group (the trivial one that contains only the identity) to the highest one  following the different group–maximal subgroup chains. There is only one space group whose point group is the identity: the space group with a single operation mod 1 in the translations, {*E*|0}.

(v)(*a*) Next, we take a point group  from list *A* whose maximal subgroup  is the trivial one and the corresponding point group operation(s) (1 or 2) chosen as coset representatives in equation (3). We construct the sets of operations as

or

for each allowed **t**_*i*_ in the list {*R*_1_|**t**_*i*_}. We check whether these operations form an allowed space group, *i.e.* it does not contain centring operations as a result of multiplication of pairs of operations. Those subsets that contain a centring operation are discarded and the remaining ones are kept as allowed subgroups . We check which groups belong to the same conjugacy class through operations of .

(v)(*b*) We now select the point groups  in list *B* which belong to the conjugacy class of the point group  in the previous step and the corresponding {*R*|**t**} operation whose point-group operation *R* fulfils equation (4). We construct the space groups whose point group is  through conjugation of the space groups  calculated in the previous step:

We keep these groups as allowed subgroups.

(vi) Once all the point groups having the identity as their maximal subgroup have been considered, in the next step we take those point groups whose chosen maximal subgroup has already been considered. We repeat step (v) for all the point groups in lists *A* and *B*. In the whole process the program keeps track of the group–subgroup relations and of the conjugacy classes. Therefore we can split all the calculated (space) subgroups into conjugacy classes and we also know which are the maximal subgroups of each group.

(vii)(*a*) The next step consists of the identification (number and symbol in *IT*A) of the space groups obtained from the point groups in list *A* and the transformation matrix to the standard setting. The space groups obtained from the point groups of list *B* having been determined through conjugation of the previous ones keep their number and symbol (unless they were conjugated by an improper operation). The transformation matrices of these groups can also be obtained through conjugation of the transformation matrices of the groups in list *A*. If the conjugation is done by an improper operation and the space group in list *A* has been identified as a member of one of the 11 enantiomorphic pairs of space groups, the conjugated space group in list *B* is its enantiomorphic partner.

(vii)(*b*) The identification of the space group is performed in a different way for each of the 32 types of point groups, depending on the number of different Bravais lattices compatible with the corresponding (and already identified) point group. First we transform the matrices of *R* to the standard description of the point group. This can involve transformation matrices whose determinant is 1, 2, 3 or 4 which, when applied to the generators {*E*|100}, {*E*|010} and {*E*|001} of the lattice, will transform the initial description in a primitive basis to a description in a centred basis with the corresponding number of centring operations. Once the pair (point group, centring) has been determined, there are a relatively small number of possible space groups to check against. The final identification involves the use of the normalizers of the point group (to check for different orientations whose rotational parts are equivalent but whose translation part is not) and general origin shifts.

(vii)(*c*) At this point we have, for each allowed subgroup, its number in *IT*A and a transformation matrix to the standard setting. Finally, we combine this matrix with the normalizers of the space group to acquire sets of possible alternative transformation matrices. Among these valid transformation matrices we select the *most appealing* one using different criteria (a rotational part as diagonal as possible, the shortest translational part *etc.*).

### A2. Filtering with respect to the Wyckoff positions

It is possible to reduce the whole list of subgroups calculated via the algorithm described in Section *A*1 when we are just interested in displacive distortions. In this case, the user must supply the occupied Wyckoff positions of the structure.

The algorithm used by *SUBGROUPS* to determine the relevant subgroups under the new restrictions is relatively simple and fast. It is based on the calculation, for each subgroup, of the total number of (positional) degrees of freedom. The number of degrees of freedom of a Wyckoff position is the number of independent parameters of the position: zero for isolated points, one for lines, two for planes and three for the general position. The total number of positional degrees of freedom of a crystal is the total number of degrees of freedom of the occupied Wyckoff positions. A given subgroup is discarded from the final list when it has the same number of degrees of freedom as at least one of its supergroups. A subgroup is kept in the list when it has more degrees of freedom than all of its supergroups.

The number of positional degrees of freedom of a given Wyckoff position is calculated in the following way. Let  represent a space group,  its point group, **w**_*i*_ with *i* = 1,…, *n*_w_ the occcupied Wyckoff positions with *n*_w_ being their total number, and  the point group isomorphic to the site-symmetry group of a representative point of **w**_*i*_. Let  be the matrices of the elements of . These matrices form a representation (in general reducible) of . The number of degrees of freedom of the Wyckoff position is the multiplicity of the identity representation in the decomposition of the representation defined by the  matrices into irreps. Geometrically, it is the dimension of the subspace kept invariant by all the matrices . Using this second point of view, we can write the condition for a vector **v** in space to be invariant under all the symmetry operations of the point group as

The number of degrees of freedom of the Wyckoff position is thus the number of linear independent equations in (8), *i.e.* the rank of the matrix  = , denoted  in the following. The total number of positional degrees of freedom  of the structure in the space group  is

The last term takes into account the number of free parameters to fix the origin, which depends only on the point group , whose elements, represented in matrix form as *R*_*j*_, form the matrix :

Note that, in general, the number *n*_w_ of occupied independent Wyckoff positions is different for different subgroups. For each subgroup, the program splits each Wyckoff position in the original subset (introduced by the user) into orbits which correspond to different Wyckoff positions in the subgroup.

Optionally, it is also possible to keep the subgroups that are attainable by lattice strains. The procedure used is exactly the same, but in the calculation of the degrees of freedom in equation (9) we must add the strain degrees of freedom of the space group, *i.e.* the number of free cell parameters not fixed by symmetry, which depends on the crystal system: one for cubic groups, two for tetragonal, trigonal and hexagonal groups, three for orthorhombic groups, four for monoclinic groups, and six for triclinic groups.

### A3. Filtering with respect to the irreducible representations

This filter can be used when the relation between the parent and the distorted structure has been introduced via the wavevector(s) option instead of the supercell option. The program shows the set of irreps of the wavevectors and the user can choose one or more irreps. *SUBGROUPS* will restrict the output to those subgroups calculated following the algorithm given in Section *A*1 which describe a distortion that transforms according to the chosen irreps. This filter can be used in combination with the filtering with respect to the Wyckoff positions. In this combination, the user can choose among the irreps which have a non-zero multiplicity in the decomposition of the mechanical representation of at least one given Wyckoff position.

For every chosen ρ irrep and every subgroup obtained without filters, the algorithm used by *SUBGROUPS* to check whether the subgroup can be realized by the action of an order parameter that transforms according to the irrep is outlined below.

First we identify the symmetry operations of the subgroup (one operation for each *R* element of the point group) expressed in the basis of the parent space group. Next we take the matrices of the irrep of the parent group *D*_ρ_(*R*), tabulated in the database *REPRESENTATIONS SG* of the BCS (Elcoro *et al.*, 2017 ▸). These matrices form a representation of the subgroup (the subduced representation into the subgroup) that, in general, is reducible into irreps of the subgroup. The chosen subgroup can be obtained though a distortion that transforms under the chosen irrep if the multiplicity of the trivial irrep is different fron zero in the decomposition of the subduced representation. In a very similar way to the approach used in the application of the filter by Wyckoff positions, we can require that a non-zero order parameter is kept invariant by all the matrices of the (subduced) representation. It is then possible to write an equation identical to equation (8) which, instead of the matrices , includes the matrices *D*_ρ_(*R*) of the irrep and where the vector **v** now represents the components of the order parameter in a space whose dimension is the dimension of the irrep. As in the preceding section [see equation (10)], we define for each subgroup the following matrix,

If the rank of the matrix for a given subgroup is higher than the corresponding rank of the matrix of any of its supergroups in the list, the subgroup is included in the final list because this means that the multiplicity of the identity irrep in the decomposition of the subduced irrep is higher in the subgroup than the corresponding multiplicity in all its supergroups. There are more degrees of freedom in the subgroup than in all its supergroups. To calculate the general order parameter that corresponds to the distortion described by the subgroup, the program takes as many linearly independent rows of *M*_ρ_ as its rank. The order parameter belongs to the subspace spanned by these vectors and can be parametrized as a linear combination of these vectors using free (unrestricted) magnitudes.

The subgroup at the bottom of the group–subgroup tree corresponds to the kernel of the irrep. The rank of *M*_ρ_ in this case is the dimension of *M*_ρ_ (the dimension of the irrep) and the order parameter is an arbitrary point in space with the dimension of the irrep.

### A4. Landau condition

When the user chooses the ‘Landau condition’ in the main input, *SUBGROUPS* lists all the subgroups that can be obtained as a result of a distortion that transforms according to a single irrep of the parent space group. It can be applied only when the user has introduced a single modulation wavevector or a set of vectors in the same star or, when the supercell option is used, if the supercell can be derived from a set of wavevectors that belong to a single star. The program identifies the set of subgroups for every irrep following the procedure explained in Section *A*3 and merges these lists into a single final list of subgroups.
